# Two linear epitopes on the SARS-CoV-2 spike protein that elicit neutralising antibodies in COVID-19 patients

**DOI:** 10.1038/s41467-020-16638-2

**Published:** 2020-06-01

**Authors:** Chek Meng Poh, Guillaume Carissimo, Bei Wang, Siti Naqiah Amrun, Cheryl Yi-Pin Lee, Rhonda Sin-Ling Chee, Siew-Wai Fong, Nicholas Kim-Wah Yeo, Wen-Hsin Lee, Anthony Torres-Ruesta, Yee-Sin Leo, Mark I-Cheng Chen, Seow-Yen Tan, Louis Yi Ann Chai, Shirin Kalimuddin, Shirley Seah Gek Kheng, Siew-Yee Thien, Barnaby Edward Young, David C. Lye, Brendon John Hanson, Cheng-I Wang, Laurent Renia, Lisa F. P. Ng

**Affiliations:** 1 Singapore Immunology Network, Agency of Science, Technology and Research, Immunos, Biopolis, Singapore, 138648 Singapore; 2 Department of Biological Science, National University of Singapore, Singapore, Singapore; 3 Department of Biochemistry, Yong Loo Lin School of Medicine, National University of Singapore, 8 Medical Drive, Singapore, 117596 Singapore; 4 National Centre for Infectious Diseases, 16 Jalan Tan Tock Seng, Singapore, 308442 Singapore; 5 Department of Infectious Diseases, Tan Tock Seng Hospital, 11 Jalan Tan Tock Seng, Singapore, 308433 Singapore; 6 Lee Kong Chian School of Medicine, Nanyang Technological University, 11 Mandalay Road, Singapore, 308232 Singapore; 7 Yong Loo Lin School of Medicine, National University of Singapore and National University Health System, 10 Medical Drive, Singapore, 117597 Singapore; 8 Saw Swee Hock School of Public Health, National University of Singapore and National University Health System, 12 Science Drive 2, #10-01, Singapore, 117549 Singapore; 9 Department of Infectious Diseases, Changi General Hospital, 2 Simei Street 3, Singapore, 529889 Singapore; 10 Department of Medicine, National University Hospital, 5 Lower Kent Ridge Road, Singapore, 119074 Singapore; 11 Department of Infectious Diseases, Singapore General Hospital, 31 Third Hospital Ave, #03-03 Bowyer Block C, Singapore, 168753 Singapore; 12 Emerging Infectious Disease Program, Duke-NUS Medical School, 8 College Road, Singapore, 169857 Singapore; 13 Biological Defence Program, DSO National Laboratories, 27 Medical Drive, Singapore, 117510 Singapore; 14 Institute of Infection, Veterinary and Ecological Sciences, University of Liverpool, Liverpool, 8 West Derby Street, Liverpool, L7 3EA United Kingdom

**Keywords:** Antibodies, Viral infection, SARS-CoV-2

## Abstract

Given the ongoing SARS-CoV-2 pandemic, identification of immunogenic targets against the coronavirus spike glycoprotein will provide crucial advances towards the development of sensitive diagnostic tools and potential vaccine candidate targets. In this study, using pools of overlapping linear B-cell peptides, we report two IgG immunodominant regions on SARS-CoV-2 spike glycoprotein that are recognised by sera from COVID-19 convalescent patients. Notably, one is specific to SARS-CoV-2, which is located in close proximity to the receptor binding domain. The other region, which is localised at the fusion peptide, could potentially function as a pan-SARS target. Functionally, antibody depletion assays demonstrate that antibodies targeting these immunodominant regions significantly alter virus neutralisation capacities. Taken together, identification and validation of these neutralising B-cell epitopes will provide insights towards the design of diagnostics and vaccine candidates against this high priority coronavirus.

## Abstract (web summary)

Characterisation of the human antibody response to SARS-CoV-2 can help the design of serological tests and vaccines. Here, the authors identify two linear epitopes in SARS-CoV-2 spike protein that elicit neutralising antibodies in several patients and could thus be useful for serology and vaccine development.

## Introduction

In December 2019, a cluster of pneumonia cases of unknown aetiology was reported in the city of Wuhan in the province of Hubei. The previously unidentified pathogen, which induces symptoms resembling an infection by the Severe Acute Respiratory Syndrome Coronavirus (SARS-CoV), was later identified as a novel coronavirus, SARS-CoV-2^1^. To date, there are more than four million laboratory-confirmed cases of human Coronavirus Disease 2019 (COVID-19), with over 280,000 deaths across 212 countries and territories (For up to date information consult https://www.who.int/emergencies/diseases/novel-coronavirus-2019/situation-reports/). After being declared a pandemic by World Health Organization (WHO) on 11th March 2020, there is a compelling need to understand and develop effective therapeutic interventions against SARS-CoV-2.

SARS-CoV-2 uses the spike (S) glycoprotein to bind to the angiotensin-converting enzyme 2 (ACE2) receptor with a better affinity than SARS-CoV S glycoprotein for entry^2^. Thus, blocking the binding to ACE2, or blocking host protease cleavage of S glycoprotein to release the fusion peptide is an efficient strategy to prevent coronavirus entry^3–5^. Several studies have assessed the immunogenicity of structural domains of recombinant SARS-CoV-2 S glycoprotein^6,7^. At the time of writing, findings on SARS-CoV-2 linear epitopes remain mostly limited to bioinformatics prediction of human B- and T-cell epitopes using SARS-CoV as a model^8–10^, and one recent pre-print described the use of a microarray of overlapping peptides to assess linear epitopes in 10 COVID-19 patients^11^. Five regions on the S glycoprotein of SARS-CoV (residues 274–306, 510–586, 587–628, 784–803, and 870-893) were predicted to be associated with a robust immune response^8^, whereas other studies reported candidate epitopes^9,10^ that require validation with patient samples.

In this study, we report the antibody profiles of COVID-19 patients and the identification of two immunodominant linear B-cell epitopes on the S glycoprotein of SARS-CoV-2. Interestingly, using S glycoprotein pseudotyped lentiviruses, we demonstrate that antibodies recognising these two linear epitopes account for a high proportion of the anti-spike response. These epitopes can potentially be used in the design of more sensitive serological assays for epidemiological or vaccine efficiency assessments.

## Results

### A spike pseudotyped lentivirus assay for virus neutralisation

**Fig. 1:**
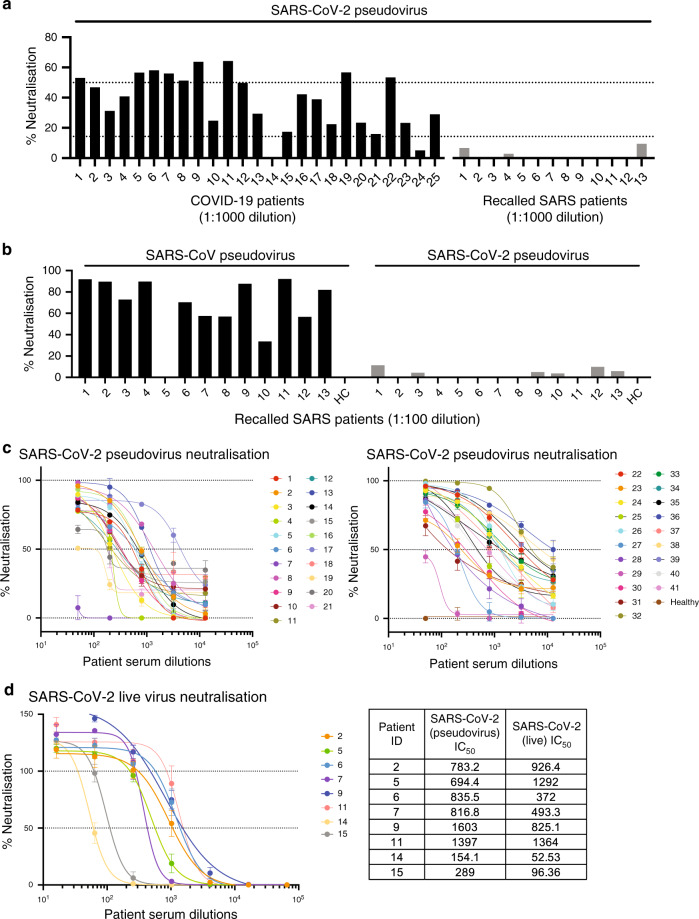
COVID-19 patient sera can neutralise pseudotyped lentiviruses expressing SARS-CoV-2 spike protein. **a** Sera of COVID-19 patients (*n* = 25) at 1:1000 dilution were incubated with luciferase expressing lentiviruses pseudotyped with SARS-CoV-2 spike (S) glycoprotein protein for 1 hour prior to infection of CHO-ACE2 cells for 48 hours. Infection levels were determined by luciferase assay, and percentage of neutralisation is presented. Recalled SARS patients (*n* = 13) and healthy controls (HC) were also conducted in parallel. Dotted lines correspond to 50% neutralisation and baseline of HC. **b** Sera of recalled SARS patients (*n* = 13, 1:100 dilution) or a healthy control (Healthy) were mixed with pseudotyped lentivirus expressing SARS-CoV or SARS-CoV-2 S glycoprotein, prior to incubation with CHO-ACE2 cells for 48 hours. Infection levels were determined by luciferase assay, and percentage neutralisation was analysed. **c** Dose–response neutralisation of pseudotyped lentivirus titration curves of COVID-19 patients (*n* = 41, 1:50 to 1:12,800 dilutions). **d** Dose–response neutralisation of live SARS-CoV-2 virus titration curves of COVID-19 patients (*n* = 8, 1:16 to 1:65,536 dilutions). Comparison table of IC_50_ values from two assays. Lines represent non-linear regression robust fit. Source data are provided as a Source Data File.

To investigate whether a biosafety level (BSL) 2 approved pseudotyped lentivirus expressing SARS-CoV-2 S glycoprotein tagged with a luciferase reporter could detect neutralising antibodies, we performed an initial screen at 1:1000 dilution using sera from 25 convalescent COVID-19 patients and from 13 SARS patients recalled in January–February 2020 as controls (Fig. 1a). Majority of the COVID-19 patients’ sera were able to neutralise >50% of SARS-CoV-2 pseudovirus entry, whereas recalled SARS patients did not show neutralisation. To validate the absence of neutralisation from the 13 recalled SARS patients, we assessed their neutralisation capacity at a lower dilution of 1:100 against the pseudotyped lentivirus expressing SARS-CoV S or SARS-CoV-2 S glycoproteins (Fig. 1b). The results indicate that these recalled SARS patients still possess antibodies specific to SARS-CoV albeit at low levels, making them an appropriate control group for subsequent linear B-cell epitope mapping.

Next, this assay was used to determine the IC_50_ values of anti-SARS-CoV-2 S-neutralising antibodies from the sera of 41 convalescent COVID-19 patients (Fig. 1c). To further validate that this safer pseudotyped lentivirus assay is representative of live SARS-CoV-2 virus neutralisation, we performed antibody neutralisation titrations for eight patients under BSL3 conditions. IC_50_ values obtained were comparable, validating the lentivirus assay (Fig. 1d). Six patients (2, 5, 6, 7, 8, 9) with sufficient amount of sera and good neutralising capacity were then selected for further characterisation. Notably, sera from these patients showed similar IC_50_ values ranging from 694 to 836, except for patient 9, who showed the strongest neutralising activity with an IC_50_ value of 1603 (Fig. 1c, Supplementary Table 4).

### Two specific linear epitopes on the SARS-CoV-2 S protein

**Fig. 2:**
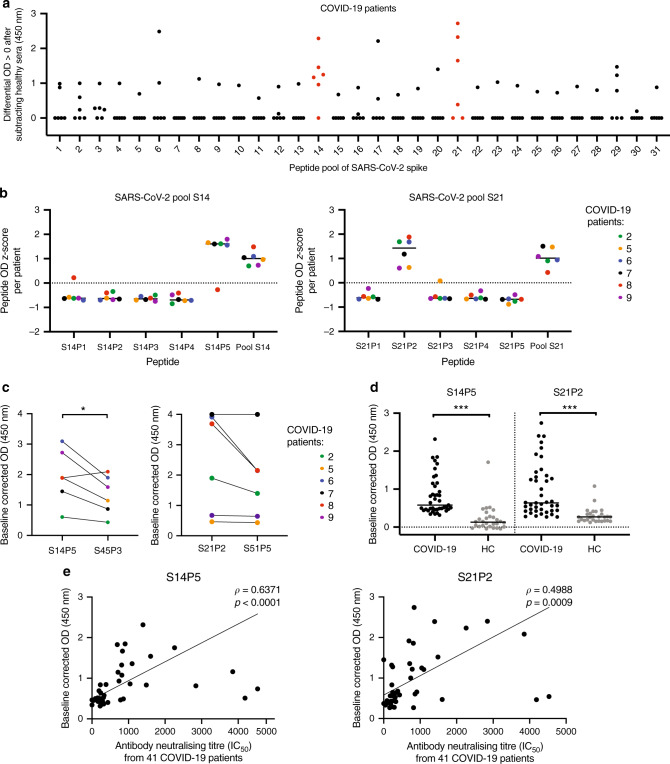
COVID-19 patient sera recognise two linear epitopes in SARS-CoV-2 spike protein. **a** Sera of COVID-19 (*n* = 6) patients at 1:1000 dilution were subjected to peptide-based IgG ELISA using peptide pools covering the entire S protein of SARS-CoV-2 in duplicates. Sera of pooled healthy donors (*n* = 13) were assessed in parallel. Data are presented as mean patient OD values subtracted of healthy control value are presented, negative values are plotted as zero. **b** Sera of COVID-19 patients (*n* = 6) were subjected to peptide-based ELISA for IgG detection using individual peptides of SARS-CoV-2 S peptide pools S14 and S21. The *z* score values of each patient were calculated using the formula [OD value of patient for peptide−average (OD values of patient)]/standard deviation (OD values of patient). Data shown are from two independent experiments and presented as mean. **c** Serum peptide binding response of COVID-19 patients on SARS-CoV-2 peptides S14P5 and S21P2, and the corresponding regions on SARS-CoV peptides S45P3 and S51P5, respectively, was determined by ELISA at 1:1000 dilution. Statistical analysis was carried out with paired parametric two-tailed *t* test (*p < 0.05). **d** Peptides S14P5 and S21P2 response in 41 COVID-19 patients and 29 healthy controls assessed by ELISA in 1% Triton X-treated plasma fraction at 1:1000 dilution. Data are presented as mean of baseline subtracted OD of two independent experiments and was analysed by Mann–Whitney *U* test (***p < 0.001). **e** Spearman correlation of ELISA response from 41 COVID-19 patients to individual peptides from **d** and sera IC_50_ neutralisation against SARS-CoV-2 S pseudotyped lentiviruses (Supplementary Table 4) were shown. Line was drawn using non-linear regression with 1/*Y*^2^ weighting. Source data are provided as a Source Data file.

We next assessed the linear antigenic targets from sera of the six selected COVID-19 patients and five recalled SARS patients using a linear B-cell peptide library spanning the entire S glycoprotein of either SARS-CoV-2 or SARS-CoV, in pools of five overlapping peptides (Fig. 2a, Supplementary Fig. 1). Interestingly, two distinct peptide pools from SARS-CoV-2 S library, pools S14 and S21, were strongly detected by sera from COVID-19 patients (Fig. 2a) and not by recalled SARS patients or healthy control serum (Supplementary Fig. 1a). Two COVID-19 patients could detect SARS-CoV S library pool S51, which partially overlaps with SARS-CoV-2 pool S21 (Fig. 2a, Supplementary Fig. 1b). This region encompasses the fusion peptide, which is highly conserved among coronaviruses^12,13^, suggesting a potential pan-SARS epitope at this location.

Further assessment of individual peptides within pools S14 and S21 narrowed down the specific region of interest to peptides S14P5 and S21P2, respectively (Fig. 2b). Recognition of S14P5 and S21P2 was stronger for the peptides of SARS-CoV-2 than on the corresponding SARS-CoV peptides (Fig. 2c). The use of these peptides as potential detection epitopes for serology assessment was further validated with 41 COVID-19 patients and 28 healthy donors (collected before the pandemic). Detection for both S14P5 and S21P2 was consistently and significantly higher in COVID-19 patients (Fig. 2d). More importantly, the level of antibodies targeting these two specific peptides determined by enzyme-linked immunosorbent assay (ELISA) significantly correlated with sera neutralising IC_50_ values (Fig. 2e), suggesting that antibodies directed at these epitopes could neutralise SARS-CoV-2.

### Antibodies against S14P5 and S21P2 can neutralise SARS-CoV-2

**Fig. 3:**
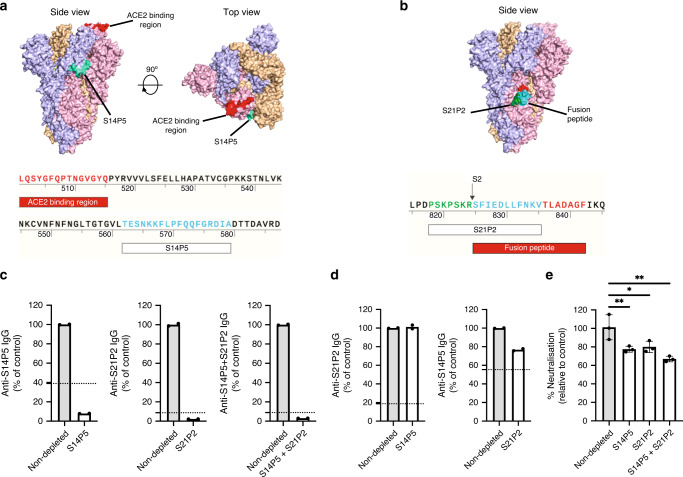
Antibodies against S14P5 and S21P2 linear B-cell epitopes neutralise SARS-CoV-2. **a**–**b** Localisation and sequences of **a** SARS-CoV-2 specific S14P5 and **b** pan-CoV S21P2 epitopes on spike (S) protein (PDB: 6VSB) are shown. Each S monomer is denoted as either pink, blue or orange. **c**–**e** Pooled sera of COVID-19 patients (*n* = 6) were added to plates coated with the corresponding peptides to deplete specific antibodies. **c**–**d** Validation of depletion by peptide-based ELISA against **c** depleted or **d** non-depleted peptides. Data of depleted sera (white bar) were normalised to percentages of the non-depleted sera (grey bar). Data are from one experiment in duplicate. Dotted line represents healthy sera mean value. **e** Non-depleted and peptide-specific antibody-depleted pooled sera were mixed with SARS-CoV-2 pseudovirus for 1 hour before infection of CHO-ACE2 cells for 48 hours. Percentage of pseudovirus neutralisation relative to the non-depleted sera, are shown. Data are presented as mean ± SD in triplicates. Statistical analysis was carried out with one-sample *t* test for each experiment (*p < 0.05, **p < 0.01). Figure is representative of two independent experiments. Source data are provided as a Source Data File.

Using a recently published structure of SARS-CoV-2 S glycoprotein in the prefusion conformation, S14P5 was shown to localise in close proximity to the receptor binding domain (RBD) (Fig. 3a), whereas S21P2 covers part of the fusion peptide (Fig. 3b). To assess the importance of these regions in controlling SARS-CoV-2 infection, antibody depletion assays were performed against S14P5 and S21P2 (Fig. 3c). Depletion efficiency and specificity were validated by ELISA, and results showed that only antibodies against the respective peptides were depleted (Fig. 3d). Interestingly, sera depleted for antibodies targeting either peptides S14P5, S21P2, or S14P5 + S21P2 significantly reduced the ability to neutralise SARS-CoV-2 pseudovirus infection, as compared with the non-depleted sera controls (Fig. 3e). Taken together, these results demonstrated that antibodies targeting these two linear epitopes account for a significant fraction of the anti-S-neutralising response.

## Discussion

In this study, we identified two immunodominant linear B-cell epitopes, S14P5 and S21P2, on the SARS-CoV-2 S glycoprotein, and further assessed the functional capacity of COVID-19 patient sera antibodies against these regions using a pseudotyped lentivirus assay. This assay uses safer third-generation lentiviruses, which will greatly benefit the scientific community in allowing rapid and safer assessments and characterisation of neutralising antibody titres in patient blood, and potential monoclonal antibodies (mAbs). Depletion assays functionally validated the positive correlation between antibody levels against these epitopes and neutralisation titres against SARS-CoV-2 pseudotyped lentiviruses. Future studies will be needed to fully understand the role and neutralisation capacity of antibodies targeting these regions.

Peptide S14P5 is localised in close proximity to the RBD. As such, it is plausible that antibodies binding to this region may sterically hinder binding to the ACE2 receptor, thereby abolishing virus infection^14^. Another possibility could be an allosteric effect on ACE2 binding. Supporting our results, the partial sequence of peptide S14P5 was computationally predicted to be immunogenic^8,10^. Peptide S21P2 partially overlaps with an epitope identified in a recent pre-print^11^ and contains a part of the fusion peptide sequence (Fig. 3b). As such, alterations to this region may potentially affect virus fusion. Indeed, targeting the SARS-CoV and MERS-CoV fusion peptide region was demonstrated to neutralise coronavirus infection with a pan-coronavirus fusion inhibitor peptide^15^.

Although our findings showed a robust IgG response against the two identified linear epitopes, it is plausible that they represent a small proportion of the total anti-S antibody response^6,7^. Nevertheless, antibody depletion assays against S14P5 or S21P2 led to >20% reduction in pseudotyped lentivirus neutralisation, validating that antibodies targeting these linear S regions are important for neutralising SARS-CoV-2 infection. Surprisingly, depletion of antibodies directed against both S14P5 and S21P2 did not significantly decrease the neutralisation as compared with single depletions, suggesting that neutralisation at these regions is not synergistic. Future studies involve the isolation of mAbs targeting these linear epitopes to allow proper quantification and comparison of peptide-specific IgG titres with antibodies directed against the RBD domain, or to other conformational epitopes. It would be interesting to also assess the level of persistence of these antibodies against linear and other conformational epitopes.

Interestingly, IgG levels against each peptide correlated positively with the patient neutralisation IC_50_ values, suggesting that quantitative serological assays against these peptides could be used as a proxy for virus exposure status as well as protection levels. However, this will require validation with other patient cohorts. Notably, the two identified epitopes present a low-to-moderate rate to impact mutations, which would minimise the possibility of false negatives in serological assays (Supplementary Table 5)^16^.

Together, these results will be essential to guide the design and evaluation of efficient and specific serological assays against linear epitopes, as well as help prioritise vaccine target designs during this unprecedented crisis.

## Methods

### Ethics statement

Written informed consent was obtained from participants in accordance with the tenets of the Declaration of Helsinki. For COVID-19 serum/plasma collection “A Multi-centred Prospective Study to Detect Novel Pathogens and Characterize Emerging Infections (The PROTECT study group)”, a domain specific review board (DSRB) evaluated the study design and protocol, which was approved under study number 2012/00917. Serum/plasma collection of recalled SARS patients “Comparison of host immune responses to coronavirus infections” was approved by DSRB under study number 2020/00091. Sera from healthy volunteers “Study of blood cell subsets and their products in models of infection, inflammation and immune regulation” was approved under study number 2017/2806.

### Patient serum and plasma fractions

Serum was collected in BD Vacutainer SST II Advance tubes (Fisher Scientific, #12927696). After clotting, serum was separated using centrifugation for 10 minutes at 1000 rcf, and aliquoted before storing at −80 °C. Patient serum was heat-inactivated for 30 minutes at 56 °C before usage for this study. Plasma fraction was harvested after 20 minutes centrifugation at 1700 rcf of blood collected in BD Vacutainer CPT tubes (BD, #362753). Plasma samples were treated by solvent/detergent treatment with a final concentration of 1% Triton X-100 (Thermo Fisher Scientific, #28314) for virus inactivation at RT for 2 hours^17^. Information on selected patients is provided in Supplementary Table 1. Patient demographics and clinical characteristics are described in Supplementary Table 2.

### Linear peptide library

The sequences used for the design of biotinylated linear peptides of the S glycoprotein of SARS-CoV and SARS-CoV-2 are under GenBank accession numbers NC_004718.3 and MN908947.3. Preliminary epitope screening was used with a library of peptides (Mimotopes, Mulgrave, VIC, Australia) consisting of 18‐mer overlapping sequences. Peptides were used individually or as pooled sets. Five to eight peptides were combined to form one pooled peptide set. Lyophilised individual peptides were dissolved in 200 μL of DMSO (Sigma‐Aldrich, #D8418-100ML) to obtain a stock solution.

### Peptide-based ELISA

B-cell linear library ELISA was performed in a similar manner to a previously established peptide-based screen^18^. In brief, streptavidin‐coated plates (Thermo Fisher Scientific, #15125) were blocked with 0.1% PBST (0.1% v/v Tween‐20, Sigma-Aldrich, #P1379-500ML, in PBS, Gibco, #20012-043) containing 1% w/v sodium caseinate (Sigma‐Aldrich, #C8654-500G, lot BCBP6469) and 1% w/v bovine serum albumin (BSA; Sigma‐Aldrich, #A7030-500G, lot SLBW5033) overnight at 4 °C, before addition of pooled or single biotinylated peptides at 1:1000 dilution in 0.1% PBST. Heat‐inactivated patient serum samples were added at 1:1000 dilution in 0.1% PBST. Horseradish peroxidase-conjugated goat anti-human IgG (H + L) antibody (Jackson ImmunoResearch, #109-035-088, lot 139159) prepared in 10% blocking buffer was used for detection of peptide‐bound antibodies. In total, 100 μL of TMB substrate (Sigma‐Aldrich, #T8665, lot SLCB5343) was used for a 5 minute development and was stopped by addition of 100 μL of 0.16 M sulfuric acid prepared from 95% to 97% Sulfuric Acid stock solution (Merck, #1.00731.1000), prior to absorbance measurements. Absorbance was measured with the following parameters: 450 nm minus 690 nm (bandwidth of 9 nm) in five flashes after a 10 second shaking at 1 mm amplitude on an Infinite M200 plate reader (Tecan, firmware V_2.02_11/06).

### Peptides S14P5 and S21P2 ELISA for 41 COVID-19 patients

Owing to the limitation of available serum samples, ELISA was performed with 1% Triton X-100 (Thermo Fisher Scientific, #28314) treated plasma fractions. ELISA was performed in similar conditions as described above with the following modifications. Nunc Maxisorp flat-bottom 96-well plates (Thermo Fisher Scientific, #442404) were coated overnight with 50 μL per well of 0.5 μg/mL of NeutrAvidin protein (Thermo Fisher Scientific, #31050). Blocking was performed for 1 hour with 0.01% polyvinyl alcohol (PVA; Sigma-Aldrich, #341584) in 0.1% PBST (blocking buffer) prepared from stock of 0.5% PVA w/v in distilled H_2_O. Peptide coating was performed at 1:2000 dilution for 1 hour. Secondary antibody was incubated for 1 hour in blocking buffer at 1:1000 dilution. Development was performed with 50 μL of TMB and stopped with 50 μL of 0.16 M sulfuric acid.

### Peptide affinity depletion of pooled sera

Using principles similar to previous work^19,20^, we performed affinity depletion as follows. Selected synthetic biotinylated peptides were added at 1:1000 dilution in 0.1% PBST to pre-blocked streptavidin-coated plates and incubated at room temperature for 1 hour. Plates were washed three times with 0.1% PBST followed by PBS wash to remove traces of Tween-20. Pooled patient sera were prepared at a dilution of 1:100 in Dulbecco’s Modified Eagle’s Medium (DMEM; HyClone, #SH30243.01, lot AE29431634), and 50 μL was added to each well and incubated for 20 minutes at room temperature for adsorption. The unbound fraction was collected after 24 rounds of adsorption. ELISA analysis was performed as described above but at 1:2000 dilution to assess the levels of peptide-specific antibodies before and after affinity depletion. Adsorbed samples were then mixed with lentiviruses pseudotyped with SARS-CoV-2 S protein as described below. Selected peptide sequences are given in Supplementary Table 3.

### Cell lines and cell culture

The human embryonic kidney epithelial cell 293T (ATCC, CRL-3216) and VERO E6 C1008 (ATCC CRL-1586 were cultured in DMEM (Hyclone, #SH30022.01) supplemented with 10% heat-inactivated foetal bovine serum (FBS; Gibco, #10270-106). A stable cell line expressing human ACE2, CHO-ACE2 (a kind gift from Professor Yee-Joo Tan, Department of Microbiology, NUS & IMCB, A*STAR, Singapore)^21^ was maintained in DMEM supplemented with 10% heat-inactivated FBS, 1% MEM non-essential amino acids solution (Gibco, #11140-050) and 0.5 mg/mL of Geneticin Selective Antibiotic (Gibco, #10131-027). Every 2–3 days, cells were passaged by dissociating the cells with StemPro Accutase Cell Dissociation Reagent (Gibco, #A1110501). ACE2 surface expression on CHO-ACE2 cells was verified using anti-human ACE2 AF647 (Santa Cruz Biotech, #sc-390851, lot B0320). Cells were routinely tested for mycoplasma contamination.

### SARS-CoV-2 and SARS-CoV pseudotyped lentivirus production

Based on the third-generation lentivirus system, pseudotyped viral particles expressing SARS-CoV or SARS-CoV-2 S proteins were produced by reverse transfection of 30 × 10^6^ of 293 T cells with 12 µg pMDLg/pRRE (Addgene, #12251), 6 µg pRSV-Rev (Addgene, #12253), 12 µg pTT5LnX-coV-SP (SARS-CoV-2 spike) or pXJ3’-S (SARS-CoV spike, a kind gift from Professor Yee-Joo Tan, Department of Microbiology, NUS & IMCB, A*STAR, Singapore)^22^ and 24 µg pHIV-Luc-ZsGreen (Addgene, #39196) using Lipofectamine 2000 transfection reagent (Invitrogen, #11668-019) and cultured in a 37 °C incubator for 3 days. Viral supernatant was harvested, spun down by centrifugation to remove cell debris and filtered through a 0.45 µm filter unit (Sartorius, #16555). Lenti-X p24 rapid titre kit (Takara Bio, #632200) was used to quantify the viral titres following the manufacturer’s instructions.

### Pseudotyped lentivirus neutralisation assay

CHO-ACE2 cells were seeded at a density of 2.5 × 10^4^ cells in 100 µL of complete medium without Geneticin in 96-well Flat Clear Bottom Black Polystyrene TC-treated Microplates (Corning, #3904). After heat-inactivation at 56 °C for 30 minutes, serially diluted patient sera were incubated in a 96-well flat-bottom cell culture plate (Costar, #3596) with an equal volume of pseudotyped virus (12 ng of p24) at the final volume of 50 μL at 37 °C for 1 h, and the mixture was added to the monolayer of pre-seeded CHO-ACE2 cells. After 1 hour of pseudotyped viral infection at 37 °C, 150 µL of culture medium was added to each well and the cells were further incubated for another 48 h. Upon removal of culture medium, cells were washed twice with sterile PBS, and then lysed in 20 µL of 1 × Passive lysis buffer (Promega, #E1941) with gentle shaking at 400 rpm at 37 °C for 30 minutes. Luciferase activity was then assessed using Luciferase Assay System (Promega, #E1510) on a Promega GloMax Luminometer.

### Live SARS-CoV-2 neutralisation assay in BSL3

Using a 96-well opaque (white) plate, 25 µL of 100 TCID_50_ of SARS-CoV-2 isolated from a naso-pharengeal swab of a patient in Singapore^23^, was incubated with 25 µL of the indicated sera dilution for 1 hour at 37 °C with 5% CO_2_. After incubation, 50 µL of 4 × 10^5^ cells/ml (VERO E6 C1008) was added into each well. The plate was subsequently incubated for 4 days at 37 °C with 5% CO_2_. Cell viability was then determined using Viral ToxGlo Assay (Promega, #G8941). In brief, 100 µL of the reagent was added into each well and incubated for 10 minutes at room temperature prior to measurement of luminescence readout using microplate reader (Tecan).

### Data visualisation and statistical analysis

Structural data of SARS-CoV-2 S protein was retrieved from Protein Databank (PDB ID: 6VSB) in homotrimeric prefusion conformation and visualised using PyMOL (Schrodinger, version 2.2.0). Data were analysed using Excel for Mac 16.16.8 and GraphPad Prism for macOS version 8.4.1. Statistical tests are indicated in the figure legends. IC_50_ values of individual patients were calculated using the [Inhibitor] vs response variable slope four parameter of GraphPad Prism, with negative values forced to zero. Correlation between pseudovirus IC_50_ and OD values were analysed using the non-parametric Spearman correlation and straight line non-linear regression robust fit functions of Prism with data from Supplementary Table 4. For the neutralisation assay with non-depleted and depleted pooled serum, one-sample *t* test for each experiment was perform to assess if the values were significantly different from 100.

### Reporting summary

Further information on research design is available in the Nature Research Reporting Summary linked to this article.

## Supplementary information

Supplementary Information

Peer Review File

Reporting Summary

## Data Availability

The source data underlying Figs. 1–3 and Supplementary Fig. 1 are provided as a Source Data file. Other data can be obtained upon reasonable request to the corresponding author. Source data are provided with this paper.

## Source data

Source Data
