# Supplementary material for: Two linear epitopes on the SARS-CoV-2 spike protein that elicit neutralising antibodies in COVID-19 patients
Source: Nat Commun. 2020 Jun 1;11:2806. doi: 10.1038/s41467-020-16638-2 (PMC7264175; doi:10.1038/s41467-020-16638-2)
Supplement: Supplementary file 1 — Supplementary Information [file 41467_2020_16638_MOESM1_ESM.pdf]

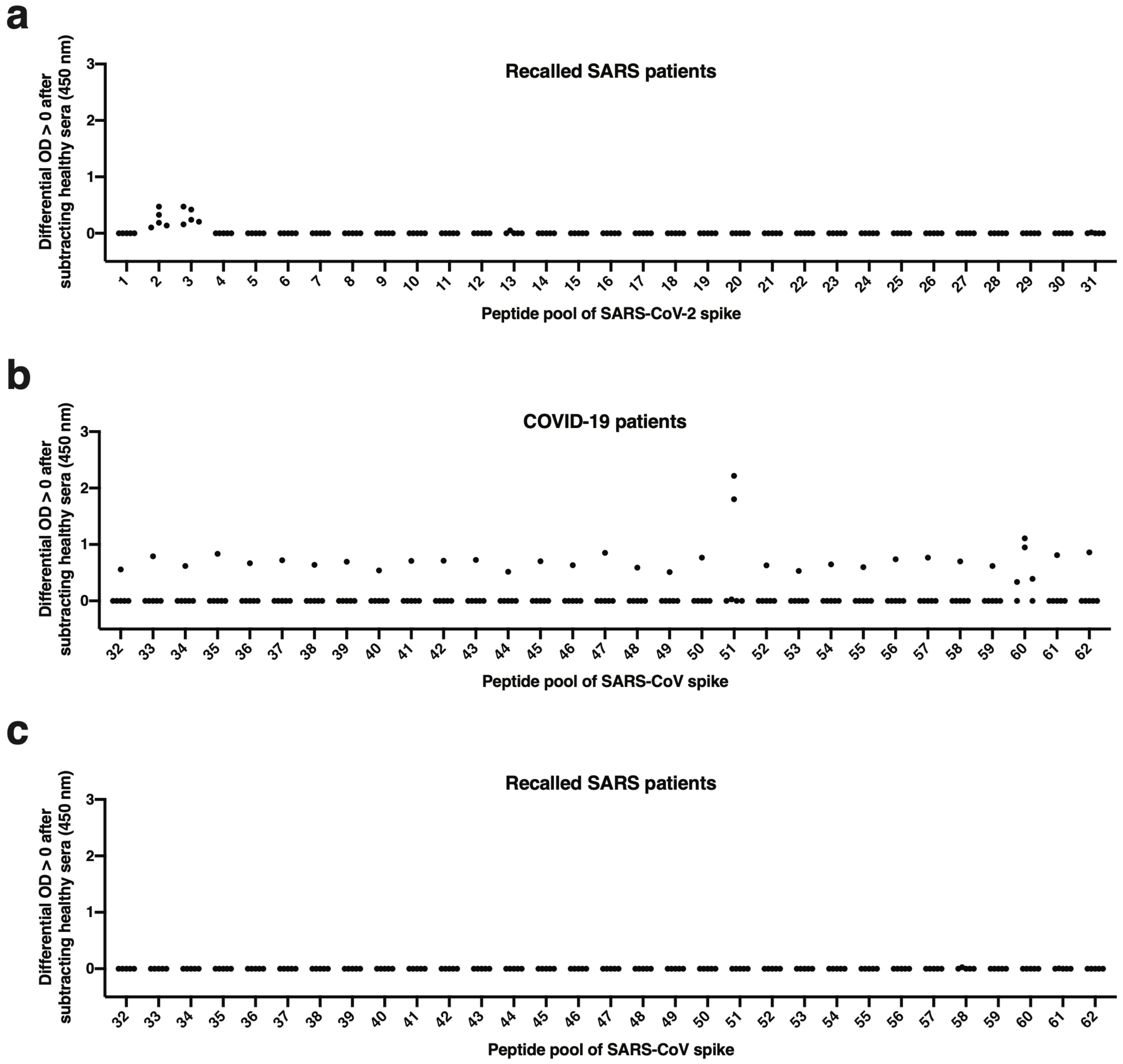

Supplementary Figure 1. ELISA screen of individual COVID-19 patient sera across the SARS-CoV-2 and SARS-CoV spike protein library. Sera of COVID-19 (n=6) and recalled SARS (n=5) patients at 1:1000 dilution were subjected to peptide-based IgG ELISA using pools of 5 peptides covering the entire S proteins of SARS-CoV-2 or SARS-CoV in duplicates. Sera of pooled healthy donors (n=13) were assessed in parallel. Data is presented as mean patient OD values subtracted of healthy control value are presented, negative values are plotted as zero. **(a)** Recalled SARS sera on SARS-CoV-2, **(b)** COVID-19 sera on SARS-CoV and **(c)** Recalled SARS sera on SARS-CoV. Source data are provided as a Source Data File.

Supplementary Table 1: Details of six COVID-19 patients individually screened against SARS-CoV-2 and SARS-CoV linear B-cell libraries

**Patient ID**	**Sex**	**Age**	**Serum collection time (days post disease onset)**
2	M	35	17
5	M	41	15
6	F	47	15
7	F	53	27
8	M	51	23
9	M	56	30

Supplementary Table 2: Demographics and clinical characteristics of 41 COVID-19 patients

**Demographics**	**Patients (n = 41)**
Age, years	43 (11)
Sex, male (%)	25 (61)
Ethnicity (Chinese)	39 (95)
Any comorbidity (%)	10 (24)
Diabetes	2 (4.9)
Hypertension	5 (12.2)
**Baseline vital signs at presentation**	
Temperature, °C	37.5 (1.0)
Heart rate, beats per minute	91.2 (18.2)
Respiratory rate, per minute	18.5 (1.9)
Systolic blood pressure, mmHg	130.6 (18.5)
Pulse oximeter oxygen saturation (%)	97.6 (2.6)
**Baseline laboratory investigations**	
White blood cells, $x10^9$/L	5.0 (1.9)
Hemoglobin, g/dL	13.8 (1.4)
Platelet, $x10^9$/L	195.3 (66.0)
Lymphocyte, $x10^9$/L	1.3 (0.6)
Neutrophil, $x10^9$/L	3.1 (1.8)
CRP, mg/L	32.0 (60.0)
LDH, U/L	497.0 (232.0)
Creatinine, µmol/L	68.2 (15.2)
ALT, U/L	34.7 (26.6)
**Outcome**	
Pneumonia with abnormal chest X ray (%)	15 (36.6)
Require supplemental oxygen (%)	8 (19.5)
ICU care (%)	4 (9.8)

Categorical variables are shown as frequency (%)
Continuous variables are shown mean (SD)
SD: Standard deviation; CRP: C-reactive protein; LDH: Lactate Dehydrogenase; ALT: Alanine Aminotransferase; ICU: intensive care unit

Supplementary Table 3: List of sequences of individual SARS-CoV-2 spike peptides tested

Pool ID	Peptide ID	Virus	Spike protein coordinates	Peptide sequence (linker)
S14	P1	SARS-CoV-2	521-570	SGSGPATVCGPKKSTNLVKNKC
S14	P2	SARS-CoV-2		SGSGKSTNLVKNKCVNFNFNGL
S14	P3	SARS-CoV-2		SGSGKCVNFNFNGLTGTGVLTE
S14	P4	SARS-CoV-2		SGSGGLTGTGVLTESNKKFLPF
S14	P5	SARS-CoV-2		SGSGTESNKKFLPFQQFGRDIA
S21	P1	SARS-CoV-2	801-850	SGSGNFSQILPDPSKPSKRSFI
S21	P2	SARS-CoV-2		SGSGPSKPSKRSFIEDLLFNKV
S21	P3	SARS-CoV-2		SGSGFIEDLLFNKVTLADAGFI
S21	P4	SARS-CoV-2		SGSGKVTLADAGFIKQYGDCLG
S21	P5	SARS-CoV-2		SGSGFIKQYGDCLGDIAARDLI
S45	P3	SARS-CoV	537-554	SGSGVLTPSSKRFQPFQQFGRD
S51	P1	SARS-CoV	761-810	SGSGREVFAQVKQMYKTPTLKY
S51	P2	SARS-CoV		SGSGQMYKTPTLKYFGGFNFSQ
S51	P3	SARS-CoV		SGSGKYFGGFNFSQILPDPLKP
S51	P4	SARS-CoV		SGSGSQILPDPLKPTKRSFIED
S51	P5	SARS-CoV		SGSGKPTKRSFIEDLLFNKVTL

Supplementary Table 4: Neutralizing IC_{50} of individual patients and their corresponding S14P5 and S21P2 baseline corrected OD values

COVID-19 Patient ID	SARS-CoV-2 (Live) IC_{50}	SARS-CoV-2 (Pseudovirus) IC_{50}	S14P5 baseline corrected OD (450nm)		S21P2 baseline corrected OD (450nm)	
			Exp1	Exp2	Exp1	Exp2
1		741.2	0.9251	0.9376	1.0029	0.9998
2	926.4	783.2	0.4532	0.4778	0.9511	1.4857
3		224.9	0.3044	0.3270	0.4857	1.1792
4		229.4	0.4518	1.2248	0.2108	0.3712
5	1204	694.4	1.8772	1.7820	1.8691	1.9659
6	372	835.5	1.6697	1.6687	2.8120	2.6706
7	493.3	816.8	1.0612	1.0909	0.2518	0.2850
8		711.4	1.2309	1.0689	1.3850	1.3262
9	825.1	1603	1.3012	1.7796	0.3837	0.5591
10		47.75	0.1054	0.8093	0.2359	0.4298
11	1364	1397	2.4507	2.1804	2.4527	2.3346
12		385.3	0.8557	0.8390	0.6623	0.6889
13		246.6	0.3260	0.3600	1.0714	1.4816
14	52.53	154.1	0.1767	0.8561	0.3008	0.4785
15	96.36	289	0.0753	0.7912	0.2069	0.3518
16		1044	0.9475	0.7728	1.1588	1.3383
17		907.7	1.8493	1.8451	0.3371	0.9745
18		245.2	0.1574	0.8234	0.6034	0.6900
19		811.5	1.6024	1.0490	1.7663	1.9503
20		4188	0.1489	0.8717	0.3710	0.5592
21		236.5	0.3116	0.9707	0.3625	0.5053
22		166.1	0.0635	0.7929	0.1852	0.3505
23		178	0.4244	0.4573	0.2293	0.8938
24		192.7	0.3336	1.0486	0.3452	0.5011
25		247.1	0.1356	0.8458	0.5373	0.5967
26		1487	0.9063	0.7614	1.4259	1.6137
27		332.7	0.2006	0.9587	0.3590	0.4799
28		217.8	0.1432	0.8547	1.2710	1.3676
29		0.5924	0.3390	0.3508	1.1595	1.7411
30		92.94	0.1585	0.8392	0.3460	0.5366

31		435.1	0.3964	0.4135	0.2671	0.9027
32		0.00002528	0.1161	0.8188	0.2915	0.4489
33		2842	0.7931	0.8352	2.2565	2.5485
34		1098	1.3473	1.3718	1.0517	1.3703
35		860	0.4906	0.4902	0.2545	0.9569
36		316	0.1629	0.8873	0.4375	0.6153
37		3851	1.2521	1.0694	1.8460	2.3201
38		4533	0.7156	0.7636	0.2046	0.8824
39		2261	1.9030	1.6008	2.2006	2.2657
40		322.2	0.3554	0.3776	0.3127	0.9613
41		189.5	0.1187	0.8282	0.2751	0.4162

Supplementary Table 5: List of mutations from a list of 10956 sequences that were curated by China National Center for Bioinformation (CNCB; https://bigd.big.ac.cn/ncov) extracted on 20 April 2020 [19] Mutations corresponding to regions in peptides S14P5 and S21P2 are highlighted in yellow.

**Peptide**	**Genome position**	**Virus number with variation**	**Annotation Type**	**Protein.Position.Amino acids change**	**Gene.Position. Codons**	**Impact Ensembl Variation - Calculated variant consequences">**	**Last Update**
	23206	2	synonymous_variant	QHD43416.1:p.548G	gene-S:c.1644ggC>ggT	LOW	2020-04-20 01:13:14
	23230	2	synonymous_variant	QHD43416.1:p.556N	gene-S:c.1668aaC>aaT	LOW	2020-04-20 01:13:14
	23242	1	synonymous_variant	QHD43416.1:p.560L	gene-S:c.1680ctG>ctT	LOW	2020-04-20 01:13:14
	23244	1	missense_variant	QHD43416.1:p.561P>L	gene-S:c.1682cCt>cTt	MODERATE	2020-04-20 01:13:14
S14P5	23248	4	synonymous_variant; coding_sequence_variant	QHD43416.1:p.562F; QHD43416.1:p.562-	gene-S:c.1686ttC>ttT; gene-S:c.1686ttC>ttY	LOW; MODIFIER	2020-04-20 01:13:14
	23266	1	missense_variant	QHD43416.1:p.568D>E	gene-S:c.1704gaC>gaA	MODERATE	2020-04-20 01:13:14
	23267	1	missense_variant	QHD43416.1:p.569I>V	gene-S:c.1705Att>Gtt	MODERATE	2020-04-20 01:13:14
	23270	1	missense_variant	QHD43416.1:p.570A>T	gene-S:c.1708Gct>Act	MODERATE	2020-04-20 01:13:14
	23271	2	missense_variant	QHD43416.1:p.570A>V	gene-S:c.1709gCt>gTt	MODERATE	2020-04-20 01:13:14

	23277	1	missense_variant	QHD43416.1:p.572T>I	gene-S:c.1715aCt>aTt	MODERATE	2020-04-20 01:13:14
	23986	1	synonymous_variant	QHD43416.1:p.808D	gene-S:c.2424gaT>gaC	LOW	2020-04-20 01:13:16
S21P2	23987	2	missense_variant	QHD43416.1:p.809P>S	gene-S:c.2425Cca>Tca	MODERATE	2020-04-20 01:13:16
	23989	1	synonymous_variant	QHD43416.1:p.809P	gene-S:c.2427ccA>ccG	LOW	2020-04-20 01:13:16
	23992	1	synonymous_variant	QHD43416.1:p.810S	gene-S:c.2430tcA>tcT	LOW	2020-04-20 01:13:16
	23996	1	missense_variant	QHD43416.1:p.812P>S	gene-S:c.2434Cca>Tca	MODERATE	2020-04-20 01:13:16
	24010	1	synonymous_variant	QHD43416.1:p.816S	gene-S:c.2448tcA>tcC	LOW	2020-04-20 01:13:16
	24011	1	missense_variant	QHD43416.1:p.817F>L	gene-S:c.2449Ttt>Ctt	MODERATE	2020-04-20 01:13:16
	24014	1	missense_variant	QHD43416.1:p.818I>V	gene-S:c.2452Att>Gtt	MODERATE	2020-04-20 01:13:16
	24023	4	missense_variant; synonymous_variant	QHD43416.1:p.821L>I; QHD43416.1:p.821L	gene-S:c.2461Cta>Ata; gene-S:c.2461Cta>Tta	MODERATE; LOW	2020-04-20 01:13:16
	24025	2	synonymous_variant	QHD43416.1:p.821L	gene-S:c.2463ctA>ctG	LOW	2020-04-20 01:13:16
	24034	98	synonymous_variant; coding_sequence_variant	QHD43416.1:p.824N; QHD43416.1:p.824-	gene-S:c.2472aaC>aaT; gene-S:c.2472aaC>aaY	LOW; MODIFIER	2020-04-20 01:13:16
	24054	25	missense_variant	QHD43416.1:p.831A>V	gene-S:c.2492gCt>gTt	MODERATE	2020-04-20 01:13:16
