# Supplementary material for: Two linear epitopes on the SARS-CoV-2 spike protein that elicit neutralising antibodies in COVID-19 patients
Source: Nat Commun. 2020 Jun 1;11:2806. doi: 10.1038/s41467-020-16638-2 (PMC7264175; doi:10.1038/s41467-020-16638-2)
Supplement: Supplementary file 3 — Reporting Summary [file 41467_2020_16638_MOESM3_ESM.pdf]

nature research

Corresponding author(s): Lisa F.P. Ng

Last updated by author(s): May 14, 2020

Reporting Summary

Nature Research wishes to improve the reproducibility of the work that we publish. This form provides structure for consistency and transparency in reporting. For further information on Nature Research policies, see Authors & Referees and the Editorial Policy Checklist .

Statistics

For all statistical analyses, confirm that the following items are present in the figure legend, table legend, main text, or Methods section.

n/a	Confirmed	
☐	☒	The exact sample size (*n*) for each experimental group/condition, given as a discrete number and unit of measurement
☐	☒	A statement on whether measurements were taken from distinct samples or whether the same sample was measured repeatedly
☐	☒	The statistical test(s) used AND whether they are one- or two-sided<br>*Only common tests should be described solely by name; describe more complex techniques in the Methods section.*
☐	☒	A description of all covariates tested
☐	☒	A description of any assumptions or corrections, such as tests of normality and adjustment for multiple comparisons
☐	☒	A full description of the statistical parameters including central tendency (e.g. means) or other basic estimates (e.g. regression coefficient) AND variation (e.g. standard deviation) or associated estimates of uncertainty (e.g. confidence intervals)
☐	☒	For null hypothesis testing, the test statistic (e.g. *F*, *t*, *r*) with confidence intervals, effect sizes, degrees of freedom and *P* value noted<br>*Give P values as exact values whenever suitable.*
☒	☐	For Bayesian analysis, information on the choice of priors and Markov chain Monte Carlo settings
☒	☐	For hierarchical and complex designs, identification of the appropriate level for tests and full reporting of outcomes
☐	☒	Estimates of effect sizes (e.g. Cohen's *d*, Pearson's *r*), indicating how they were calculated

Our web collection on statistics for biologists contains articles on many of the points above.

Software and code

Policy information about availability of computer code

Data collection	When appropriate data was collected using excel. For ELISA readings data was collected on Infinite M200 plate reader (Tecan, firmware V_2.02_11/06). For luminescence, data was collected with GloMax plate reader (Promega).
Data analysis	Structural data of SARS-CoV-2 Spike protein was retrieved from Protein Databank (PDB IDs 6VSB) in homotrimeric prefusion conformation and visualised using PyMOL (Schrodinger, version 2.2.0). Data was analyzed using GraphPad Prism versions 7.03 to 8.3.0 (GraphPad Software, San Diego, CA, USA) and excel for Mac version 16.16.8 (Microsoft, USA)

For manuscripts utilizing custom algorithms or software that are central to the research but not yet described in published literature, software must be made available to editors/reviewers. We strongly encourage code deposition in a community repository (e.g. GitHub). See the Nature Research guidelines for submitting code & software for further information.

Data

Policy information about availability of data

All manuscripts must include a data availability statement. This statement should provide the following information, where applicable:

- Accession codes, unique identifiers, or web links for publicly available datasets
- A list of figures that have associated raw data
- A description of any restrictions on data availability

All raw data is available upon reasonable request from the corresponding author.

Field-specific reporting

Please select the one below that is the best fit for your research. If you are not sure, read the appropriate sections before making your selection.

Life sciences study design

All studies must disclose on these points even when the disclosure is negative.

Sample size	Initial screen was performed on 25 serum samples from 25 individual COVID-19 patients and 13 individual serum from SARS patients recalled for this study. All subsequent analysis was performed on 6 selected serums from 6 individual patients due to good neutralization and serum volume availability. For individual identified peptides, experiments were performed on 41 plasma samples from individual patients. Live virus and pseudovirus titration curves were performed on 8 and 41 individual patient sera respectively.
Data exclusions	No data was excluded
Replication	All assays were performed in technical duplicate or triplicate. Neutralization assays were performed once for initial screen (one previous screen was performed with a different cell line that was a lot less permissive to lentiviral expression (results were comparable and not shown). Depletion assays were performed twice. All ELISA experiments were performed twice independently.
Randomization	Serums were allocated in the experimental groups depending on their confirmed infections with SARS-CoV and SARS-CoV-2
Blinding	Neutralization assays were performed blind by an independent researcher with numbered serum tubes. Individual peptides coating for ELISA and depletions were coated by one researcher, and an other researcher performed the experiment without knowing the peptides coated on the plates.

Reporting for specific materials, systems and methods

We require information from authors about some types of materials, experimental systems and methods used in many studies. Here, indicate whether each material, system or method listed is relevant to your study. If you are not sure if a list item applies to your research, read the appropriate section before selecting a response.

Materials & experimental systems

n/a	Involved in the study	
☐	☒	Antibodies
☐	☒	Eukaryotic cell lines
☒	☐	Palaeontology
☒	☐	Animals and other organisms
☐	☒	Human research participants
☐	☒	Clinical data

Methods

n/a	Involved in the study	
☒	☐	ChIP-seq
☒	☐	Flow cytometry
☒	☐	MRI-based neuroimaging

Antibodies

Antibodies used	HRP-conjugated goat anti-human IgG (H+L) antibody (Jackson ImmunoResearch, # 109-035-088, lot 139159)<br>anti-human ACE2 AF647 (Santa Cruz Biotech, sc-390851, lot B0320)
Validation	We routinely use these antibodies in our laboratory. One positive control (Chikungunya E2EP3 peptide and positive patient serum was used as positive and normalization control on each ELISA plate) and multiple negative control (each peptide/pool with no serum, sera without peptide and well as blank control) in duplicate is used on each plate to validate all ELISA antibody and substrates.

Eukaryotic cell lines

Policy information about cell lines

Cell line source(s)	HEK293T ATCC-CRL-3216<br>CHO-ACE2. CHO-K1 (ATCC-CCL61) cell line modified to express ACE2 from a plasmid containing G418 resistance gene (10.1128/JVI.80.2.941–950.2006). ACE2 expression on CHO cells was verified by flow cytometry using anti-human ACE2 (Santa Cruz Biotech, sc-390851, lot B0320) on LSRII instrument (BD) using FACS Diva software (not shown in manuscript).
Authentication	Cell lines were authenticated by the provider
Mycoplasma contamination	Cell lines were routinely tested for mycoplasma contamination.
Commonly misidentified lines<br>(See ICLAC register)	*Name any commonly misidentified cell lines used in the study and provide a rationale for their use.*

Human research participants

Policy information about studies involving human research participants

Population characteristics	The 6 individual patients analyzed in detail in this study, are comprised of 4 males 2 females who recovered from COVID-19. Age ranges from 35 to 56, and serum collection varied from 15 to 30 days post symptoms onset. Details are give in the methods section.
Recruitment	Written informed consent was obtained from participants in accordance with the tenets of the Declaration of Helsinki. Patients were recruited after testing positive for SARS-CoV-2 or SARS-CoV as described in the methods.
Ethics oversight	For COVID-19 serum collection "A Multi-centred Prospective Study to Detect Novel Pathogens and Characterize Emerging Infections (The PROTECT study group)", a domain specific review board (DSRB) evaluated the study design and protocol, which was approved under study number 2012/00917. Serum collection of SARS recall "Comparison of host immune responses to coronavirus infections" (ref) was approved by DSRB under study number 2020/00091. Sera from healthy volunteers "Study of blood cell subsets and their products in models of infection, inflammation and immune regulation" was approved under study number 2017/2512.

Note that full information on the approval of the study protocol must also be provided in the manuscript.

Clinical data

Policy information about clinical studies

All manuscripts should comply with the ICMJEguidelines for publication of clinical research and a completedCONSORT checklist must be included with all submissions.

Clinical trial registration	NA
Study protocol	NA
Data collection	NA
Outcomes	NA
