# Supplementary material for: Two linear epitopes on the SARS-CoV-2 spike protein that elicit neutralising antibodies in COVID-19 patients
Source: Nat Commun. 2020 Jun 1;11:2806. doi: 10.1038/s41467-020-16638-2 (PMC7264175; doi:10.1038/s41467-020-16638-2)
Supplement: Supplementary file 2 — Peer Review File [file 41467_2020_16638_MOESM2_ESM.pdf]

Editorial Note: This manuscript has been previously reviewed at another journal that is not operating a transparent peer review scheme. The manuscript was considered suitable for publication without further review at Nature Communications.
